# Integrative analysis of the Pekin duck (*Anas anas*) MicroRNAome during feather follicle development

**DOI:** 10.1186/s12861-017-0153-1

**Published:** 2017-07-20

**Authors:** Xingyong Chen, Kai Ge, Min Wang, Cheng Zhang, Zhaoyu Geng

**Affiliations:** College of Animal Science and Technology, Anhui Agricultural University, Hefei, People’s Republic of China

**Keywords:** Peking duck, MicroRNAome, Feather, Lipid synthesis and metabolism

## Abstract

**Background:**

The quality and yield of duck feathers are very important economic traits that might be controlled by miRNA regulation. The aim of the present study was to investigate the mechanism underlying the crosstalk between individual miRNAs and the activity of signaling pathways that control the growth of duck feathers during different periods. We therefore conducted a comprehensive investigation using Solexa sequencing technology on the Pekin duck microRNAome over six stages of feather development at days 11, 15, and 20 of embryonic development (during the hatching period), and at 1 day and 4 and 10 weeks posthatch.

**Results:**

There were a total of 354 known miRNAs and 129 novel candidate miRNAs found based on comparisons with known miRNAs in the *Gallus gallus* miRBase. The series of miRNAs related to feather follicle formation as summarized in the present study showed two expression patterns, with primary follicle developed during embryonic stage and secondary follicle developed mainly at early post hatch stage. Analysis of miRNA expression profiles identified 18 highly expressed miRNAs, which might be directly responsible for regulation of feather development. The Kyoto Encyclopedia of Genes and Genomes (KEGG) pathway analysis suggested that in addition to Wnt and transforming growth factor (TGFβ) signaling pathways, which were widely reported in response to follicle formation, another group of signaling pathways that regulate lipid synthesis and metabolism, such as the phosphatidylinositol signaling system and glycerolipid metabolism and signaling, are also responsible for follicle formation.

**Conclusion:**

The highly expressed miRNAs provide a valuable reference for further investigation into the functional miRNAs important for feather development. Lipid synthesis and metabolism related signaling pathways might be responsible for lipid formation on the surface of feather, and should be paid much more attention for their relation to feather quality.

**Electronic supplementary material:**

The online version of this article (doi:10.1186/s12861-017-0153-1) contains supplementary material, which is available to authorized users.

## Background

The structure of bird skin and feathers differ from that of similar structures in mammals [1]. Flight feathers and down feathers serve different functions, and the quality and yield of down feathers are very important economic traits [2]. The feathers of ducks are widely used for decoration and to line clothing, bedding, and so on [3]. Fast-growing Pekin ducks usually reach market weight around 35–40 d of age [4, 5], when the plumage is still far from maturity [6]. This inevitably reduces the value of the feathers at this stage. To maximize the economic potential of duck feathers, the diversity that is evident in feather morphology should be used as an advantage to gain deeper insight on the genomic and epigenetic levels. With recent advances in epigenetics, the present is the opportune time to develop our understanding of morphogen signals in feather follicles at the genomic level. Increasing evidence suggests that miRNAs serve as biological regulators that mediate gene expression [7].

Strands of miRNA exert observable impact on target genes of vertebrates and invertebrates and show hallmarks of incorporation into endogenous regulatory networks. Yuan et al. found 399 conserved miRNAs that played roles in the regulation of hair follicle cycling in cashmere goats (*Capra hircus*) [8]. The most highly expressed miRNA in that study was quite different from that observed in duck feather regeneration [9]. In addition, miRNA expression exhibits significant differences between embryonic stages and feather regeneration stages in the chicken and duck, respectfully. Bao et al. (2016) reported that 226 miRNA genes differentially expressed among three embryonic stages in the chicken in which 21 miRNAs targeted genes were feather related [10]. However, none of the differentially expressed miRNAs were found during feather regeneration in duck [9].

In our previous study, genes that regulated feather regeneration differed significantly from those associated with similar functions in mammals [11]. Thus, we assumed that feather development in avian species is regulated by miRNA, which might differ from that in mammalian skin and hair. Therefore, we selected skin samples of ducks from the early embryonic stages up until feathers were fully developed at 10 weeks of age. In addition, we investigated the miRNAome during feather development using Solexa sequencing. A better understanding of the complex mechanisms underlying feather development in ducks might be of benefit to the down feather industry and genes participating in the signaling pathways that regulate feather follicle formation might be used as candidate genes for molecular marker screening in the future.

## Methods

### Ethics statement

All animal procedures were performed in accordance with guidelines developed by the China Council on Animal, Care and protocols were approved by the Animal Care and Use Committee of Anhui Agricultural University, China.

### Sample preparation

From our previous study on follicle development in broilers and geese, we deduced that follicle formation in the duck starts around day 11 of embryonic development (11EM), progresses so that the body becomes lightly covered with feathers by day 15 (15EM), following which feathers completely cover the body at day 20 (20EM) [12]. A significant increase in secondary follicle growth has been observed post hatch [12]. After 4–5 weeks of age, changes have been observed in duckling feathers, which become fully mature feathers at 10 weeks of age [13].

White Pekin duck embryos (Anhui Taiyang Poultry Co. Ltd., China) of similar genetic background were selected at days 11, 15, and 20 of embryonic development (11EM, 15EM, and 20EM). Ducks were reared under normal conditions of light and temperature and had free access to food and water at Anhui Taiyang Poultry Co. Ltd., China. From 1 day to 10 weeks of age, ducks were fed with commercial diets that met all National Research Council requirements (NRC, 1994). Ducks were slaughtered by sectioning the neck 2–4 h after the last meal at three stages of development: 1 d, 4 and 10 weeks of age (1 DB, 4WK, and 10WK) post-hatch. For each stage, six skin samples collected from an area just above the second to third thoracic vertebrae were used for total RNA extraction. Each of these samples was immediately stored in a tube with 1 mL RNALater (Qiagen) and stored at −20 °C. Another six skin samples from the same area were stored immediately in vials containing 10% buffered formalin for 24 h and transferred to 70% ethanol for storage until embedding and processing. Tissue sections were laterally cut for Masson trichrome staining [12]. The analysis of the slides was performed according to Chen et al. [12].

### RNA isolation

Total RNA was extracted from the skin samples using a mirVanamiRNA Isolation Kit (Ambion, USA) according to Qin et al. (2013) [14]. The purified RNA yield was determined by the absorbance at 260 nm with an ND-2000 NanoDrop spectrophotometer (Thermo Fisher, USA), and RNA quality was evaluated with the BioAnalyzer 2100 system (Agilent Technologies, USA) that was done at Shanghai Biotechnology Corporation.

### Small RNA library preparation and deep sequencing

RNA smaller than 200 bp was enriched with the mirVanamiRNA isolation kit (Ambion). RNA was then precipitated with ethanol and dissolved in water. Small RNAs had linkers ligated to them and bar-coded cDNAs were prepared using a TruSeq Small RNA Sample Prep Kit (Illumina, USA) following the manufacturer’s instructions. Small RNA (1 μg) was ligated with adapters at the 3′ and 5′ ends. Adapter-ligated RNA was reverse-transcribed with SuperScript II Reverse Transcriptase (Invitrogen, USA), and then PCR-amplified (11 cycles). Individual libraries were analyzed on a BioAnalyzer (Agilent) for the presence of linked cDNA at the appropriate size (140–150 bp), as determined by the BioAnalyzer. The amplified cDNA constructs were then purified from agarose gel in preparation for sequencing analysis, which was conducted at the Shanghai Biotechnology Corporation, using the Illumina HiSeq 2500 System (Illumina, CA, USA) according to the manufacturer’s instructions.

### Bioinformatic analyses of sequencing data

The small RNA sequence reads were pre-processed using the FASTX-Toolkit [15], excluding low-quality reads (ambiguous N, quality <10 nucleotides [nt], and length < 18 nt) as well as the 3′ and 5′ adapters and poly (A) sequences. Further annotation analyses were performed using the commercial software CLC Genomic Workbench 5.5. The resulting clean reads were aligned against various databases, including ncRNA, piRNA, and Rfam, allowing a maximum mismatch of 2 nt to remove noncoding RNA, such as rRNA, tRNA, snRNA, and snoRNA. The remaining sequences were analyzed by a BLAST *Gallus gallus* search against the Sanger miRBase (version 21.0). Reads that did not match any of the databases mentioned above were marked as non-annotated. Non-annotated sequences were searched against the *Gallus gallus* genome using the miRCat program included in the sRNAToolkit (http://srna-workbench.cmp.uea.ac.uk/tools/analysis-tools/mircat/). Using default settings, 100 nt flanking each side of the genomic sequences were extracted for prediction of RNA secondary structure using RNAfold [16]. Only typical stem-loop hairpin structures with free energy lower than −20 kcal/mol were considered potential novel miRNAs. After the completion of all annotation steps, sequencing libraries were used for size distribution and saturation analysis. All sequence data have been submitted to the NCBI Sequence Read Archive (https://www.ncbi.nlm.nih.gov/geo/query/acc.cgi?acc=GSE101542) under accession No. SRA073195.

### Identification of miRNA differential expression

We first normalized miRNA-sequence data from 18 libraries as transcripts per million (TPM). The followingnormalization formula was used:

Normalized expression = Actual miRNA count/Total count of clean reads × 1000,000. If the normalized expression of a given miRNA was zero, its expression value was modified to 0.01. Normalized sequence counts were used to perform a one-way ANOVA to determine significant differences. The expression ofa specific miRNA was considered significantly different if the *p*-value and False Discovery Rate (FDR) value were both less than 0.05. FDR indicates the expected proportion of false positives among the results deemed significant [17].

### Time series analysis

The Short Time-series Expression Miner (STEMv 1.1) program was used to cluster and visualize possible profiles and changes in expression over time (less than 8 time points) in differentially expressed (DE) miRNAs. The maximum unit change in model profiles between time points was adjusted to 1 and the maximum number of model profiles to 50. MicroRNA expression profiles were clustered according to correlation coefficients. Statistical significance of the number of genes assigned to each profile versus the numbers expected were computed by an algorithm and the default *p*-value was 1e-5 [18]. Statistically significant model profiles that were similar to each other were grouped together to form clusters of profiles.

### Prediction and analysis of miRNA target genes

As there is no appropriate database or method to predict duck miRNA target genes, miRanda version 3.1 (http://www.microrna.org/microrna/getMirnaForm) was used for target gene prediction. To completely assess functions of the differentially expressed miRNAs, we conducted gene ontology (GO) and KEGG pathway analyses enriched with predicted miRNA targets. The analyses were conducted using the fuzzy clustering algorithm” to reduce redundancy among functionally related pathways that share similar target genes. Terms with Benjamini-corrected enrichment *p* < 0.01 and FDR < 0.05 were considered. An association map was generated that summarizes the enriched pathways in a graphical representation of the relationships between terms based on the similarity of their target genes. All of the data analysis methods used in this experiment were the same as mentioned in Qin et al. [14] because we both performed the miRNA sequencing at Shanghai Biotechnology Corporation.

### MiRNA validation via stem-loop RT-PCR

Small RNAs (< 200 nt) were isolated using the mirVanamiRNA Isolation Kit (Ambion, USA) following the manufacturer’s instructions. Quantitative real-time PCR (qRT-PCR) was carried out as previously described in an ABI PRISM 7500 Fast Realtime PCR System (Ambion, USA), using the SYBR Premix Ex Taq™ Kit (TaKaRa, Japan) [19]. The reactions were carried out in a volume of 20 μL, containing 2 μL of diluted cDNA, 200 nM of each primer, and 16 μLPCR Master Mix under the following conditions: 95 °C for 30 s; 45 cycles at 95 °C for 5 s; 58 °C for 15 s; and 72 °C for 20 s. A thermal denaturing cycle then followed at 95 °C for 15 s and 60 °C for 1 min applied to determine the dissociation curves, which were used to verify the specificity of PCR amplifications. All reactions were run in triplicate for each sample. Ten miRNAs, including seven conserved and three novel miRNAs were validated, and 5.8S rRNA was selected as a reference gene for normalization (Additional file 1: Table S1). The experimental data was analyzed using the 2^-∆∆CT^method.

## Results

### Microscopic observation of follicle characters

**Fig. 1:**
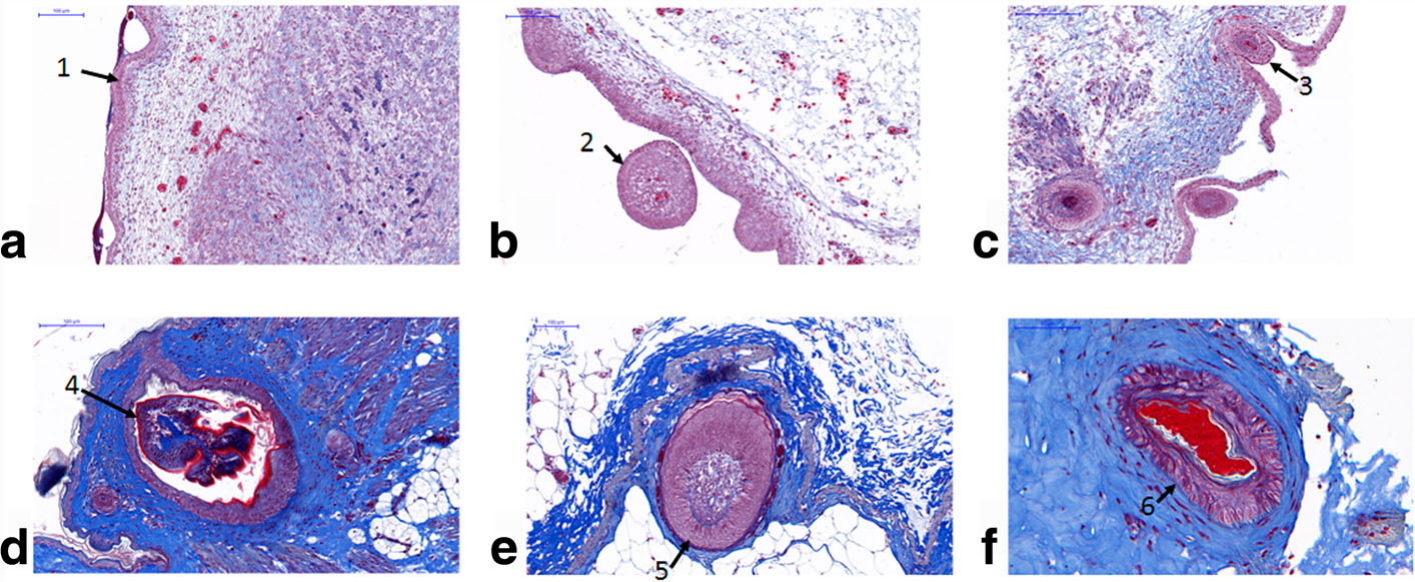
Masson trichrome staining of feather follicle. **a** 11EM; **b** 15EM; **c** 20EM; **d** 1 DB, **e** 4WK; **f** 10WK. 1. Cell proliferation to form the feather bud; 2. feather sheath; 3. a single layer of follicle; 4. pulp and vessel; 5. thickened epithelium; 6. keratinized barb ridge

At the early embryonic stage of 11EM, cell proliferation was detected to form feather bud at epithelium (Fig. 1a). At 15EM, the primary follicle formed from the epithelial layer, and also feather sheath filled in newly formed follicle (Fig. 1b). At 20EM, the follicle and feather sheath were closely linked together to form a single layer (Fig. 1c). After incubation, primary follicle formed and filled with pulp and vessel was detected in birds at 1 day old (Fig. 1d). At 4 weeks of age, epithelium thickens around the dermal papilla, and cell proliferation is high in the basal layer of the epithelium (Fig. 1e). At 10 weeks of age, the barbs and barbules keratinized and lack active barb ridge formation (Fig. 1f).

### Overview of Solexa-sequencing of feather follicle small RNAs

**Fig. 2:**
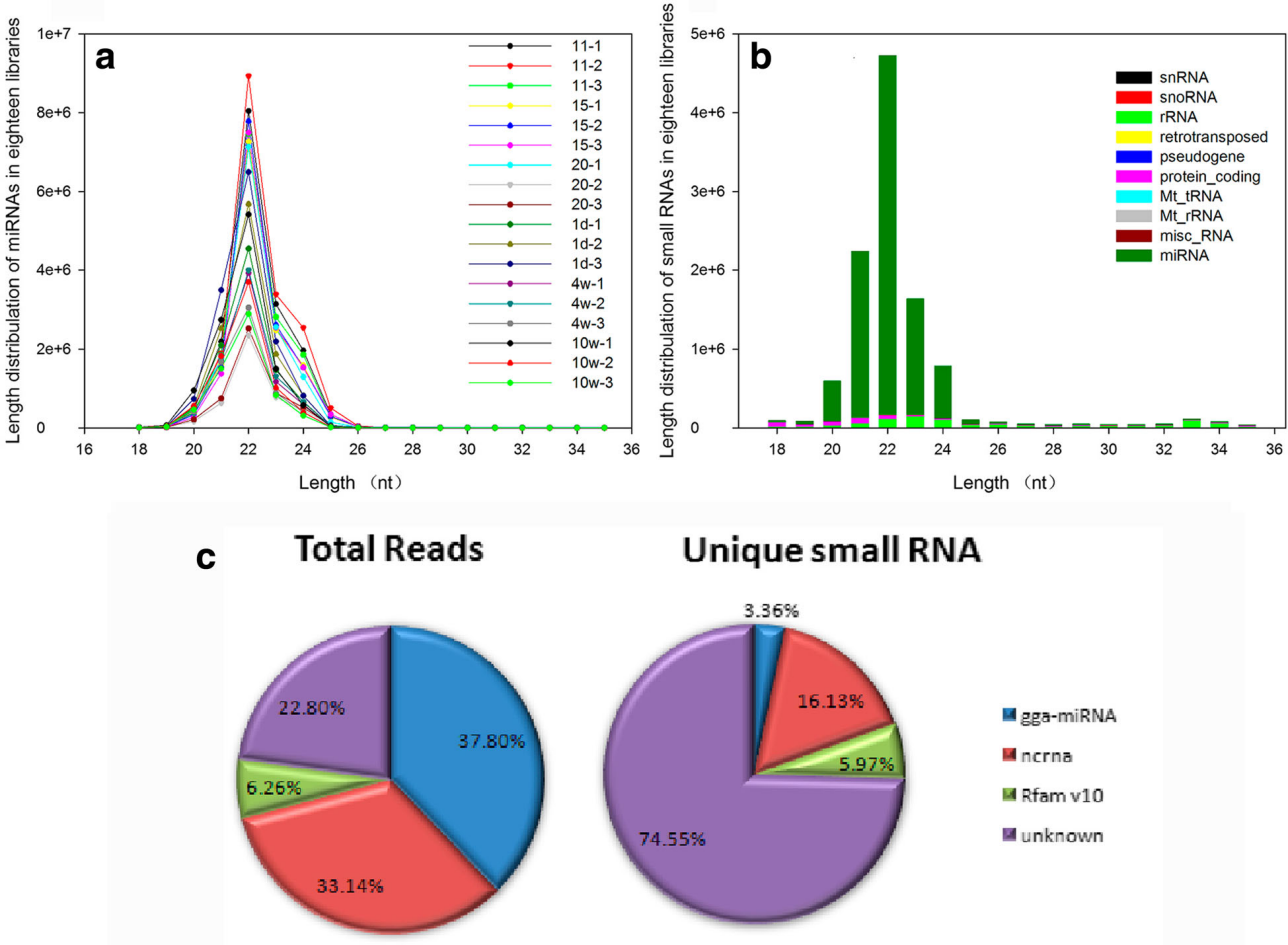
Basic analysis of sequenced data. **a**-**b** Sequence length distribution of known miRNAs (**a**) and small RNAs (**b**); **c** Count distribution of total reads (*left*) and unique small RNAs (*right*)

There was a total of 18 libraries (three in each stage) representing six stages of feather development. A total of 461,981,782 raw reads, and 443,133,642 clean reads after removing the adapters and discarding sequences either shorter than 17 nt or longer than 35 nt (Additional file 2: Table S2), were obtained from the 18 libraries. The clean distinct tags (unique sRNAs) in all samples ranged from 71.5–85.7% in each library; these tags were retained by clustering of similar reads and alignment to the *Gallus gallus* miRNA database (Additional file 2: Table S2). Furthermore, the number of new sequences observed for known small RNAs and duck miRNAs (found in the miRBase) reached a plateau when the number of sequenced reads was 17,000,000, suggesting that the library capacity approached saturation. Similar plots of the other 17 libraries are presented in Additional file 3: Figure S1 As to the sequences distribution of the small RNA, the most abundant size class was 22 nt, (50.34%), followed by 23 nt (17.08%), 21 nt (16.86%), and 24 nt (9.45%), in that order (Fig. 2a). All mapped clean reads were further annotated and classified by alignment against non-coding RNAs (ncRNAs) in the Rfam database and miRbase 21 in order to further assess the efficiency of Solexa sequencing for miRNA detection as exhibited in Fig. 2b. Except for the enriched miRNA sequence, there were also some other kinds of ncRNAs, such as rRNAs, tRNAs, mRNAs, snoRNAs, and other sRNAs that added the diversity of the Rfam database (Fig. 2b). The annotated small RNAs that can be matched to chicken miRNA (gga-miRNAs) accounted for 37.8% of the total sequence reads, and only 3.36% of unique sequence reads within the sequenced 18 libraries (Fig. 2c).

### Identification of potential novel miRNAs

To detect additional potential miRNAs, unannotated sequences within 18 to 35 nt were searched against chicken miRNA database and analyzed using the miRDeep software. Sequenced reads that did not match any known ncRNA, piRNA, and miRNAs from the Rfam database, and had typical stem-loop hairpin structures with free energy lower than −20 Kcal/mol were considered potential novel miRNA. There were totally 129 candidate novel miRNAs were predicted based on novel miRNA identification criterion and then named as gga-miR-new-N (1–129) (Additional file 4: Table S3). The most frequent sequence length was 18 nt (27.9%), followed by those that measured 22 nt (17.1%), 19 and 20 nt (15.5% in each), and 23 nt (9.3%).

### Expression analysis of miRNAs and miRNA families in duck feather development

A total of 354 miRNAs were identified in duck feather follicles (Additional file 5: Table S4). All miRNAs were expressed at varying levels, ranging from 1 to more than 100,000,000 reads. Mullokandov et al. (2012) developed a Sensor-seq to show that over 60% of detected miRNAs had no discernible activity and only the most highly expressed miRNAs in a cell mediate target suppression [20]. From the normalized miRNAs reads, there were 296 miRNAs with mean expression values below 1000 Transcript Per Million (TPM) (243 below 100 and 53 between 100 to 1000); these miRNAs account for almost 83.6% of the total number of miRNAs (68.6% and 15.0%, respectively) within the 18 libraries (Additional file 5: Table S4). The mean number of miRNAs with expression values over 1000 TPM was 58 (16.4%), among which 19 miRNAs (5.4%) expressed over 10,000 TPM, and two miRNAs expressed over 100,000 TPM, and the 19 top most expressed miRNAs accounted for 82.5% of the total unique miRNAs (Additional file 6: Figure S2 and Additional file 7: Table S5).

From the 129 novel miRNAs detected in 18 libraries, the total reads were at a relatively low level. There were 100 novel miRNAs expressed less than 100 times, and 17 were at 100–1000 TPM (of which four were sequenced more than 500 times), 12 were at 1000–10,000 times, and only gga-mir-new-12 was sequenced more than 10,000 times (Additional file 4: Table S3).

### Identification of differentially expressed miRNAs at different developmental stages

MicroRNA with high expression in a certain developmental stage might play important roles in regulating the biological processes. Thus, we compared the differentially expressed (DE) miRNA between two successive selected sequencing stages of the 354 mature miRNAs. In total, 205 DE miRNAs (fold change ≥2; *p* < 0.05) were identified during feather formation, and 91 DE miRNAs were identified with a *p* < 0.01 (Table 1). There were 73 DE miRNAs (*p* < 0.01) during incubation period, and there were 18 DE miRNA were detected before 4WK (*p* < 0.01) and no DE miRNA detected between 4WK and 10WK (*p* < 0.01).

**Table 1:** Numbers of differentially expressed miRNAs between libraries

Numbers of differentially expressed miRNAs						
Comparison between adjacent libraries	Total DE miRNAs		Total DE miRNAs-up		Total DE miRNAs-down	
	0.01	0.05	0.01	0.05	0.01	0.05
EM11/EM15	24	54	18	33	6	21
EM15/EM20	18	37	2	7	16	30
EM20/1 DB	31	73	13	27	18	47
1 DB/4WK	18	39	12	23	6	16
4WK/10WK	0	2	0	1	0	1

*DE* Differentially expressed (*p* < 0.05 or *p* < 0.01, log2 fold change >2); up, up-regulation; down, down-regulation

**Fig. 3:**
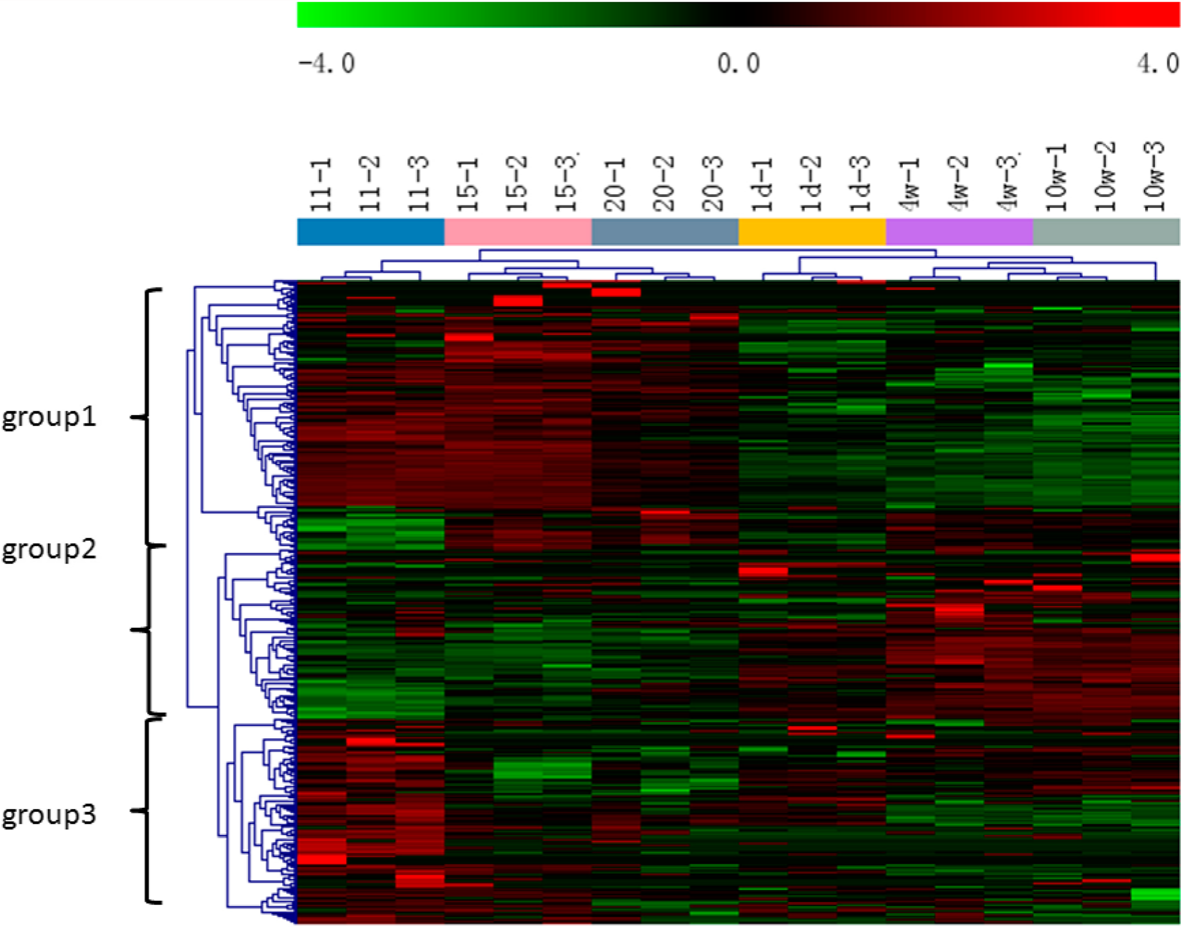
Heatmap visualization of miRNA with significant expression variance in response to different stages of feather follicle development. Samples are represented in the column, miRNAs are represented in rows, and the color scale on top illustrates the relative expression levels of indicated miRNA across all samples: *red* denotes expression >0 and *green* denotes expression <0

**Fig. 4:**
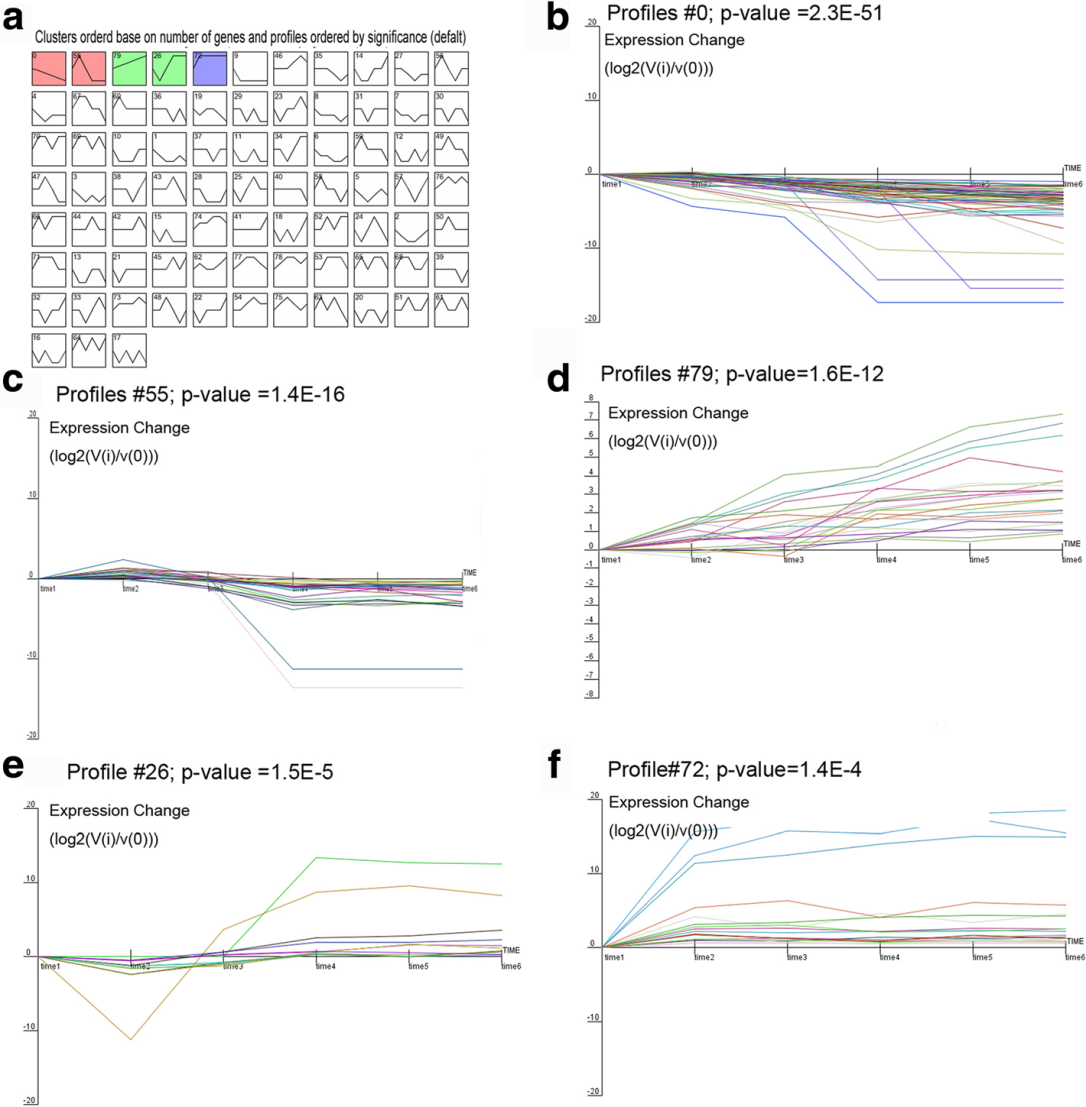
Clustering of miRNA expression profiles over the entire experimental period. Each box corresponded to a model of expression profile and only colored profiles reached statistical significance were listed. **a** The total clustering of miRNA into each expression profile, and five colored profiles were listed as profile #0 (**b**), profile #55 (**c**), profile #79 (**d**), profile #26 (**e**), and profile #72 (**f**)

**Fig. 5:**
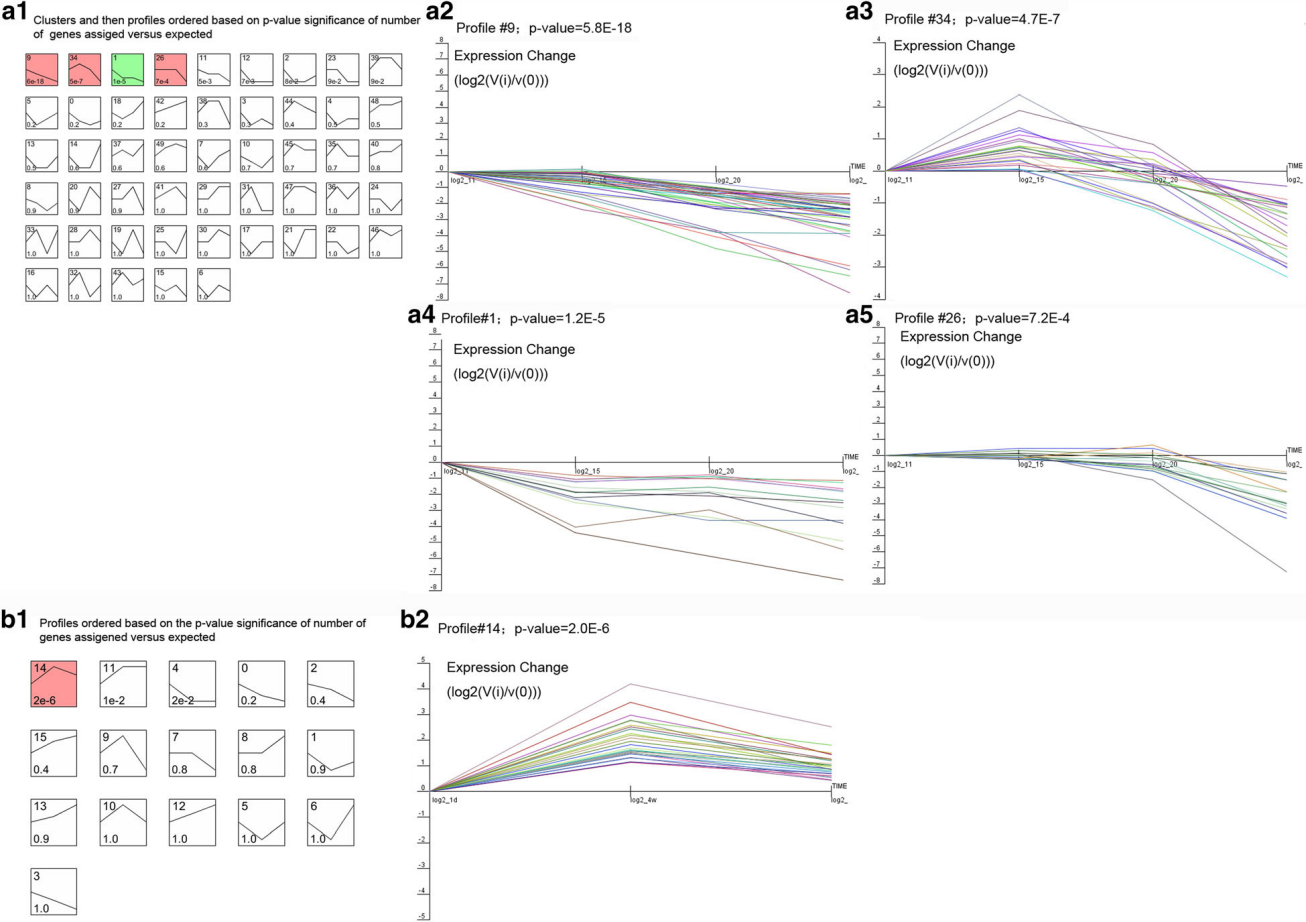
Clustering of miRNA expression profiles during two stages of feather follicle development. During the first stage (11EM to 1 DB), there were four profiles (profile #9, profile #34, profile #1, and profile #26) found statistically significant as shown in A2, A3, A4, and A5, respectively. In the second stage (1 DB to 10WK), only one profile (profile #14) was found statistically significant expression as shown in B2

From the heatmap generated (Fig. 3), we can first divide genes into two major groups with the bottom (larger) group separated into two groups. Therefore, the genes could be divided into three groups. In groups 1 and 3, a cluster of genes was highly expressed from the period of embryonic development until day 20 of incubation, with low expression during the period of growth from day one to 10 weeks of age. In addition, another group of genes inversely related to groups 1 and 3, showed low expression during incubation and high expression during periods of growth. In the present study, STEM (Short Time-series Expression Miner) was used to cluster and visualize possible changes in the profiles of DE miRNAs at six time points of feather development. A total of 133 of the 205 DE miRNAs (64.9%) (Additional file 8: Table S6) were observed during feather formation. These miRNAs were significantly clustered into five expression patterns (Fig. 4, the default *p*-value was 1e-4). Out of 80 possible clusters, the miRNA expression profiles of three were found to be significant; these are presented in Fig. 4. The cardinality of each cluster ranged from 13 DE miRNAs in cluster #26, to 55 in cluster #0 (Additional file 9: Table S7). Visual examination of these clusters suggested that up- and down-regulated DE miRNAs originated from the EM15 time point. In cluster #26, DE miRNAs were first down-regulated and then up-regulated at EM15, then reached a peak at the 1 DB time point. In cluster #0, DE miRNAs showed a gradual decrease and remained stable at the 1 DB time point. In cluster #72, DE miRNAs exhibited a slight, gradual increase. These findings might again suggest that primary follicle formation is almost completed before birth. Thus, we compared the expression patterns of genes from 11EM to 20EM, as well as from 1 day to 10 weeks of age using STEM. A total of 83 out of 291 DE miRNAs observed from 11EM to 20EM were significantly clustered into four expression patterns (Fig. 5), whereas 28 out of 165 DE miRNAs observed from 1 day to 10 weeks were significantly clustered into a single expression pattern (Fig. 5).

### MicroRNA target predictions and KEGG Orthology analysis

**Fig. 6:**
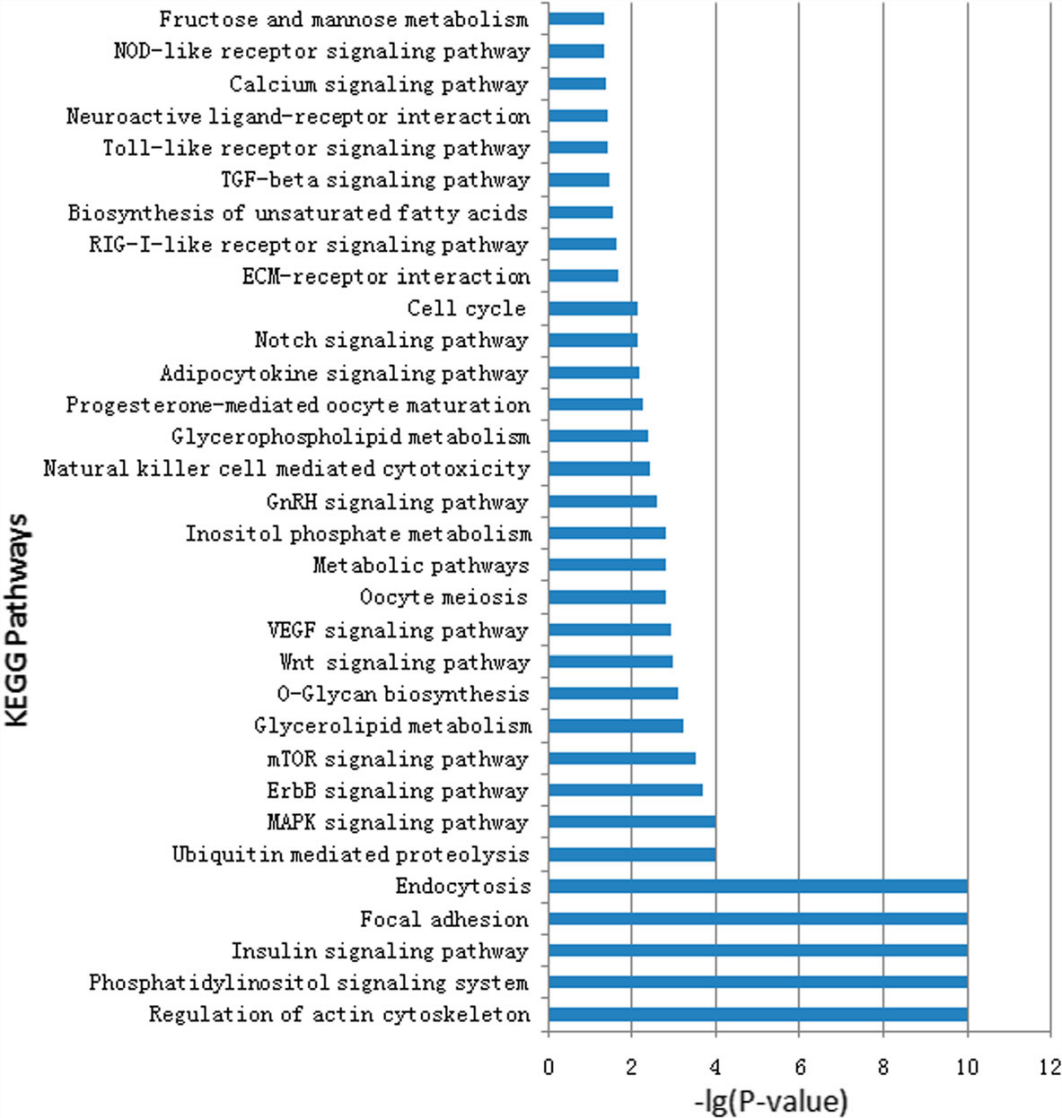
KEGG Orthology analysis of the most abundant miRNAs. Significantly enriched KEGG pathways (32) were identified using the target genes of the 18 most abundant miRNAs in 18 libraries

The miRanda version 3.1 was used to predict the target genes of the identified miRNAs. In this analysis, only the 18 most abundant miRNAs (excluding the let-1 family, which is ubiquitously expressed, but including miR-140) were used for target gene prediction and a total of 1253 genes were detected and then subjected for KEGG Orthology analysis (Additional file 10: Table S8). There were 32 significantly enriched pathways selected according to *p* value (*p* < 0.05) (Fig. 6). The highly expressed miRNAs were involved in regulation of the actin cytoskeleton, focal adhesion, ubiquitin-mediated proteolysis, and MAPK, ErbB, mTOR, Wnt, Notch, and TGF-β signaling pathways, in which Wnt, Notch, and TGF-β are closely related to feather formation and regeneration [21–23]. In addition, some pathways related to lipid metabolism, such as phosphatidylinositol, O-glycan biosynthesis, inositol phosphate, and glycerophospholipid metabolism, were actively regulated by miRNAs in skin tissue. These findings could be attributed to the fact that lipids cover the surfaces of waterfowl feathers, thereby conferring water-repellent properties.

### Validation of sequencing data

**Fig. 7:**
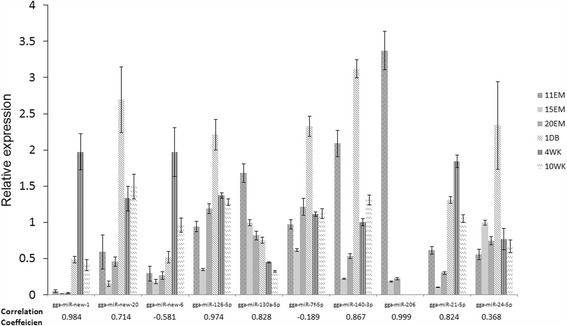
Expression profiles of candidate miRNAs during feather development and correlations with Solexa sequencing results

To verify Solexa sequencing data, seven miRNAs in known databases and three from novel miRNAs with varying expression levels were selected. QRT-PCR was conducted for all developmental stages. Stem-loop RT-PCR primers are presented in Additional file 1: Table S1. The Pearson’s correlation coefficients (r-values) of real-time PCR and Solexa sequencing were calculated as −0.190 and −0.581, respectively. The other eight r-values ranged from 0.368–0.999, indicating high consistency with previous results (Fig. 7).

## Discussion

### Time points selection for miRNA libraries construction

Some miRNAs are involved in the development of hair follicles and skin, and are related to hair follicle cycles in mammals [8, 24, 25]. However, there have been few studies on poultry miRNAs and their relation to the development of feather growth. Avian skin and feathers are very different from mammalian skin and hair, because of the differences in function (despite similarities in structure) between flight and down feathers. Zhang et al. detected 96 miRNAs in duck skin during feather regeneration at 8 weeks of age [9]. However, feather growth might already have been initiated during the early embryonic stages. Thus, we chose the six time points to elucidate the miRNA expression profiles of duck feather formation in this experiment.

### MicroRNA database is reliable for analysis of miRNA expression profiles during duck feather growth

We identified 354 known gga-miRNAs and 129 potential novel miRNAs between the embryonic stages and 10 weeks of age. In viewing the sequenced reads, the libraries capacity approached saturation demonstrated that the deep sequencing data were able to represent the miRNA transcriptome profiles of duck feather growth. Among the annotated small RNAs, only one third were matched to chicken miRNA, suggesting that the miRNAs including very richful diversities, and the aforementioned results promote confidence in the deep sequencing data and the present data are reliable for analysis of miRNA expression profiles.

### The highly expressed miRNA were mostly related to regulation of cell cycle

Skin development is governed by complex processes of gene activation and silencing, and entails miRNA-dependent modulation of gene expression. Many miRNAs that regulate hair follicle formation are also involved in the regulation and development of cancer and skin diseases in mice and humans, mainly because they regulate cell cycles. MiR-214 regulates skin morphogenesis and hair follicle cycling by targeting β-catenin [26, 27]. It can also be expressed in several human tumors, such as those of ovarian cancer and breast cancer, by targeting Bim to promote cell proliferation. However, few studies have focused on feather formation in birds, let alone in waterfowl.

There were only 19 miRNAs expressed over 10,000 TPM suggested that the majority of abundantly expressed miRNAs were fewer in number. We also found that more than half of the 19 miRNAs were related to regulation of the cell cycle. The most abundant miRNAs were gga-miR-10a and gga-miR-10b, which represented more than 2,000,000 TPM within 18 libraries. MiR-10a reportedly plays an important role in regulation of squamous cell growth in the neck area [28]. The miRNAs miR-10a and miR-10b are close homologs, differing only by a single central nucleotide [29]. In the mouse embryo, miR-10a is mainly expressed in a region of the posterior trunk [30]. The miRNAs miR-10a and miR-10b are reportedly involved in cell proliferation and regulation of the cell cycle [31]. Two other miRNAs, gga-miR-26a and gga-miR-181a, were highly expressed and ranked as the third and fourth most highly expressed miRNAs, respectively. They represented more than 1000,000 TPM in 18 libraries and have been identified to play a role in tissue regeneration via the BMP/SMAD1 signaling pathway [32, 33]. Frucht et al. reported that miR-181a plays a key role in regeneration of basilar papillae in the cochlea of the chicken [32]. Leeper et al. also demonstrated that miR-26a could inhibit cellular differentiation and apoptosis by altering the TGFβ signaling pathway [34]. It has been widely accepted that BMP and TGFβ signaling pathways play pivotal roles in feather follicle growth and development. MiR-205, the seventh most highly expressed miRNA, targets the lipid phosphatase SHIP2 in epithelial cells, whereas the corneal-specific miR-184 interferes with the ability of miR-205 to suppress SHIP2 levels [35]. MiR-21, the 11th most highly expressed miRNA, negatively regulates BMP4 and has been reported in the epidermis and hair follicle epithelium of normal mouse skin [36]. Other miRNAs, which were responsible for feather growth andregulation of gene expression, showed significantly lower levels of expression. Expression of miR-24 alters the normal process of hair keratinocyte differentiation, leading to altered expression of differentiation markers, by direct repression of the hair keratinocyte stemness regulator TCF-3, which sustains postnatal epidermal homeostasis [37]. MiR-31 is responsible for the anagen phase of the follicle cycle and could alter hair shaft formation anddifferentiation in hair matrix keratinocytes [25]. MiR-31 negatively regulates expression of Fgf10, components of the Wnt and BMP signaling pathways, including sclerostin, BAMBI, and Dlx3 transcription factor, as well as selected keratin genes. MiR-203 is a skin- and keratinocyte-specific miRNA that has been implicated in repression of ‘stemness’ in epidermal progenitors by targeting p63 and the suppressor of cytokine signaling-3 (SOCS-3) [38]. Up-regulation of miR-203 is required for differentiation in human keratinocytes and is dependent on the activation of the Pkc/AP-1 pathway [39]. Thus, it is clear that the 19 most highly expressed miRNAs might regulate feather generation via BMP and TGFβ pathways.

Comparing the 19 most highly expressed miRNAs with the total miRNA expression during the period of feather regeneration in ducks [9], only miR-30a, miR-126, and miR-181a were also highly expressed during feather regeneration in ducks [40]. A single specific stage of feather growth cannot clearly illustrate miRNA function, because its expression pattern is both tissue- and developmental stage-specific. However, we propose that these three miRNAs are mainly responsible for feather growth, whereas other miRNAs might also share responsibility for feather and follicle growth.

### Differentially expressed miRNA might suggest the different feather developmental stage

Based on the differentially expressed miRNAs at different developmental stages, and also combined with previous studies on feather growth [12], we speculated that the peak time for primary feather follicle formation might be during the incubation period, while the peak for second feather follicle formation was during the early growth period post hatch and before 4 weeks of age. However, we could not conclude the concise growth period for the secondary feather follicle growth since we did not construct the miRNA database from one day post hatch till 4 weeks of age. Only two genes detected in this experiment played a role in feather formation after 4 weeks of age. However, the feathers of ducklings are normally replaced around 4 weeks of age, suggesting no obvious changes in structure and morphology in the follicles of white Pekin duck after this period.

In birds, skin and feathers serve different functions, as the flight and down feathers are adapted for different roles. Follicle formation is initiated during the early embryonic stage [41]. From the most highly expressed miRNAs, we found miR-10b and miR-205a were also found to be differentially expressed at the eighth embryonic day in chicken, which targeted genes ALDH1A3 and GALGA_5AKI_KRT19, respectively [10]. However, feather growth differs from that of follicular formation [42]. Of the most highly expressed miRNAs during follicle formation, we found miR-181a and miR-30a were also the most highly expressed during feather regeneration in duck [9]. The involvement of mmu-miR-199a and miR-140 in hair follicle and skin development are related to hair follicle cycling in mammals [24, 43]. These two miRNAs were down-regulated from 11EM to 1 DB, suggesting their role in the regulation of feather generation during the early embryonic periods. Only two DE miRNAs were detected from 4WK to 10WK: miR2131–3p and miR-196-1-3p, which reportedly targets the 3′ untranslated region (UTR) of the *rarab* gene, and is responsible for pectoral fin bud initiation in zebrafish [44].

### The KEGG signaling pathway analysis

Signal transduction pathways and molecules that control hair and follicle formation in mammals have been well understood over the past few decades. However, the signals and molecules that control avian feather growth and regeneration might be quite different because of the complexity of ectodermal organs with hierarchical branching patterns, that regulate endothermy, communication, and flight [1].

In order to evaluate the differentially expressed miRNAs, the KEGG signaling pathways were further analyzed. The major pathways predicted in the present study have been previously reported. Feather formation signal pathways comprised mainly the regulation of the actin cytoskeleton, phosphatidylinositol signaling system, insulin signaling pathway, focal adhesion, endocytosis, ubiquitin-mediated proteolysis, MAPK, ErbB, mTOR, and glycerolipid metabolism, Wnt, VEGF, Notch, the cell cycle, and TGF-β, among others. Most of these signal pathways have already implicated in other tissue regeneration processes, such as skin and hair follicle development in the mouse, human, and cashmere goats [8, 45–48]. However, it is worth mentioning, that with the exception of the signal pathways that are usually reported, there were other types of signal pathways that regulated lipid synthesis and metabolism, such as the phosphatidylinositol signaling system, glycerolipid metabolism, O-glycan biosynthesis, inositol phosphate metabolism, glycerophospholipid metabolism, and biosynthesis of unsaturated fatty acids. A layer of fat and oil typically coats the surface of feathers in waterfowl [49]. A certain amount of fat and oil is necessary to maintain the water repellent properties of such feathers. Thus, we propose that genes that were involved in lipid synthesis and metabolism signal pathways could be candidate genes that are also responsible for feather quality.

## Conclusions

We first sequenced the miRNAome in duck skin from its embryonic day till market size. The highly expressed miRNAs founded provide a valuable reference for further investigation into the functional miRNAs important for feather development. Feather follicle development could be divided into two distinct stages, one for primary follicle and the other for secondary follicle, which initiated different miRNAs expression. Lipid synthesis and metabolism related signaling pathways might responsible for feather quality.

## Additional files

Additional file 1: Table S1. Annotations of sequenced miRNAs. Table S1–1 Number of small RNA reads. Table S1–2 Summary of reads matching noncoding RNA. Table S1–3 Summary of small RNA matching noncoding RNA databases. (XLS 286 kb) (XLS 24 kb)

Additional file 2: Table S2. Predicted chromosomal positions and counts of novel miRNAs. (XLS 86 kb)

Additional file 3: Figure S1. Saturation plots of 18 libraries. (TIFF 1306 kb)

Additional file 4: Table S3. Summary of reads distribution of 354 known duck miRNAs. Table S4–1 Expression profiles of 354 miRNA in 18 libraries. Table S4–2 Raw reads distribution. Table S4–3 The distribution of numbers for normalized miRNAs. (XLS 90 kb)

Additional file 5: Table S4. Summary of top 20 most abundant miRNAs in 18 libraries. (XLS 362 kb)

Additional file 6: Figure S2. Counts characteristics of the unique miRNAs in each library. Starting from the miRNA with the highest counts (x-axis), the black bar represents the accumulative proportion of miRNAs in total counts of each library. The red horizontal line represents the proportion of individual miRNA versus the total 354 miRNAs. (TIFF 3097 kb)

Additional file 7: Table S5. Expression profiles of DE miRNAs during the whole duck feather follicle development. (XLS 33 kb)

Additional file 8: Table S6. Information about the five significant clusters. (XLS 97 kb)

Additional file 9: Table S7. Target genes of the top expressed 18 miRNAs. (XLS 35 kb)

Additional file 10: Table S8. Primer information for miRNA RT-qPCR. (XLS 286 kb)
